# Protective Effects of Polydatin on Septic Lung Injury in Mice via Upregulation of HO-1

**DOI:** 10.1155/2013/354087

**Published:** 2013-01-30

**Authors:** Xiao-hui Li, Xia Gong, Li Zhang, Rong Jiang, Hong-zhong Li, Meng-jiao Wu, Jing-yuan Wan

**Affiliations:** ^1^Chongqing Key Laboratory of Biochemistry and Molecular Pharmacology, Chongqing Medical University, Chongqing 400016, China; ^2^Department of Anatamy, Chongqing Medical University, Chongqing 400016, China; ^3^Department of Pathophysiology, Chongqing Medical University, Chongqing 400016, China; ^4^Laboratory of Stem Cell and Tissue Engineering, Chongqing Medical University, Chongqing 400016, China

## Abstract

The present study was carried out to investigate the effects and mechanisms of polydatin (PD) in septic mice. The model of cecal ligation and puncture (CLP-)induced sepsis was employed. Pretreatment of mice with PD (15, 45, and 100 mg/kg) dose-dependently reduced sepsis-induced mortality and lung injury, as indicated by alleviated lung pathological changes and infiltration of proteins and leukocytes. In addition, PD inhibited CLP-induced serum tumor necrosis factor-**α** (TNF-**α**) and interleukin-6 (IL-6) production, lung cyclooxygenase-2 (COX-2) and inducible nitric oxide synthase isoform (iNOS) protein expressions and NF-**κ**B activation. Notably, PD upregulated the expression and activity of heme oxygenase (HO-)1 in lung tissue of septic mice. Further, the protective effects of PD on sepsis were abrogated by ZnPP IX, a specific HO-1 inhibitor. These findings indicated that PD might be an effective antisepsis drug.

## 1. Introduction

Acute lung injury (ALI), induced by pneumonia, sepsis, multiple trauma, aspiration of gastric contents and severe burns, is a critical illness syndrome marked by excessive production of proinflammatory mediators, massive infiltration of leukocyte, and rapid alveolar injury, resulting in respiratory failure and high short-term mortality [1, 2]. Sepsis is a common and life-threatening medical condition, with more than 50% of patients admitted to an intensive care unit (ICU) developing ALI [2]. Despite recent advances in antibiotic therapy and intensive care, the prognosis of ALI remains poor. Hence, prevention of sepsis and septic complications is urgent. 

Septic lung injury in mice is associated with excessive production of proinflammatory mediators including tumor necrosis factor-*α* (TNF-*α*), interleukin-6 (IL-6), cyclooxygenase-2 (COX-2), and inducible nitric oxide synthase (iNOS) [3]. Interfering with proinflammatory cytokines to relieve lung injury has been widely applied, while less attention was paid to anti-inflammatory molecules. Previous studies have demonstrated that upregulating anti-inflammatory molecules can significantly attenuate sepsis-induced inflammatory response [3, 4]. Heme oxygenase (HO-1), also called heat shock protein 32 (hsp32), is an inducible enzyme catalyzed rate-limiting step in the oxidative degradation of heme to free iron, biliverdin, and carbon monoxide [5]. Recent findings showed that HO-1 and its subsequent metabolites exerted anti-inflammatory and antiapoptotic activities. Overexpression of HO-1 exhibited a blunted activation of NF-*κ*B and less production of cytokines induced by LPS [6, 7]. Therefore, specific induction of HO-1 expression may be a new target in the treatment of septic lung injury. 

**Figure 1:**
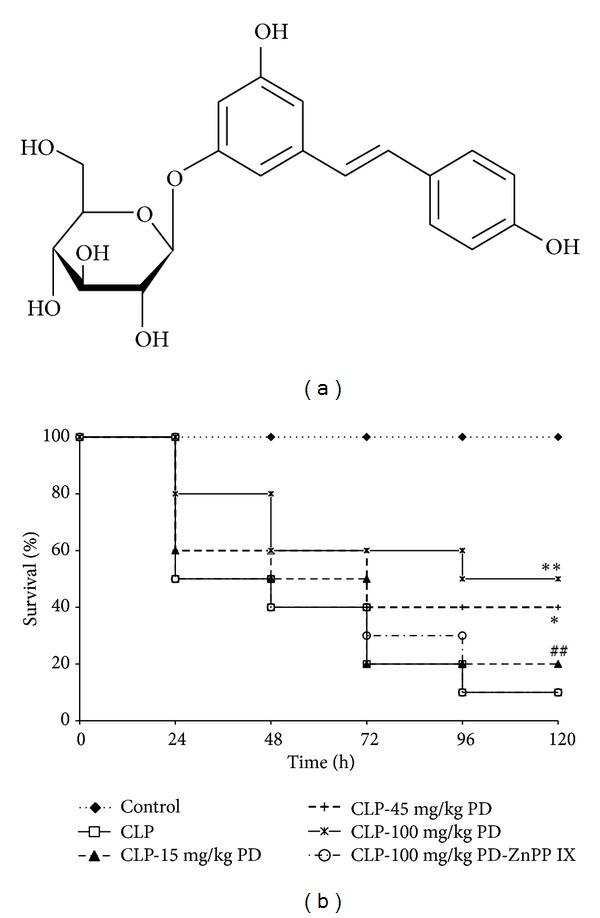
Effects of PD on survival rates in mice. (a) Chemical structure of PD. (b) Survival rates (*n* = 10). Mice were pretreated intraperitoneally (i.p.) with vehicle (PBS) or PD (15, 45, and 100 mg/kg, resp.) in the presence or absence of ZnPP IX (40 mg/kg) 1 h prior to CLP. Survival rate of mice was monitored for 48 h.  *P < 0.05,  **P < 0.01 versus the CLP group.  ^##^ *P* < 0.01 versus the CLP-PD group.

Polydatin (3, 4′, 5-trihydroxystibene-3-*β*-mono-D-glucoside, PD, Figure 1(a)), the glycoside of resveratrol, is a crystal component extracted from the root stem of the perennial herbage* Polygonum Cuspidatum* Sieb.et Zucc, which is traditionally used for patients with chronic bronchitis, hepatitis, and shock [8]. Previous studies indicated that PD had various activities such as inhibiting the platelet aggregation, lowering the level of blood lipid, reducing lipid peroxidation, dilating blood vessels, and protecting from myocardial ischemia/reperfusion damage [9–11]. A number of studies have reported that PD and its derivatives have anti-inflammatory activities [8, 9]. However, the efficacy, as well as the mechanisms of action of PD on acute lung injury, remains unknown. 

On the basis of these reported results, we used a typical septic-induced ALI model, cecal ligation and puncture procedure, to examine the effects of PD on septic acute lung injury and to further study its possible molecular mechanisms.

## 2. Materials and Methods

### 2.1. Animals

Mice weighing 18~22 g were obtained from the Experimental Animal Center of Chongqing Medical University. The animals were kept in individual wire-bottom cages. All animals were fed with a standard laboratory diet and water ad libitum. They were maintained in a controlled environment at a temperature of 20–25°C and 50 ± 5% relative humidity under a 12 h dark/light cycle, and were acclimatized for at least l week before use. All experimental procedures involving animals were approved by the Animal Care and Use Committee of Chongqing Medical University. 

### 2.2. Reagents

PD (C_20_H_22_O_8_, MW: 390.40, purity ≥ 95%) was purchased from Nanjing Debiochem Co. Ltd. (Nanjing, China). ZnPP IX was obtained from Sigma (St. Louis, MO, USA). Bicinchoninic acid (BCA) protein assay kit was purchased from Pierce (Rockford, IL, USA). TNF-*α* and IL-6 enzyme-linked immunosorbent assay (ELISA) kits were obtained from the Bender MedSystems (Vienna, Austria). Rabbit anti-COX-2, iNOS, HO-1, and HO-2 antibodies were obtained from Abcam (Cambridge, MA, UK). *β*-actin antibodies were purchased from Cell Signaling Technology (Boston, MA, USA). TransAM NF-*κ*B transcription factor assay kit was purchased from Active Motif (Carlsbad, USA). 

### 2.3. Experimental Protocols

The CLP protocol was performed as previously described [7, 12]. Under sodium pentobarbital (30 mg/kg, i.p.) anesthesia, a 1-cm long midline incision was made and the cecum was exposed. The cecum was ligated by silk 4-0 and punctured twice with an 18-gauge needle, and then the cecum was gently squeezed to extrude a small amount of feces. The cecum was repositioned, after which the abdomen was closed in two layers with 4-0 silk thread. Finally, 1 mL of saline was administered subcutaneously for resuscitation. Control operated mice underwent the same surgical procedures, but the cecum was neither ligated nor punctured. After surgery, animals were put back to their home cages with free access to water. 

Mice were randomly divided into six groups: control group, CLP group, CLP-PD (15 mg/kg) group, CLP-PD (45 mg/kg) group, CLP-PD (100 mg/kg) group, and CLP-PD-ZnPP IX group. The control and the CLP groups were pretreated with an intraperitoneal (i.p.) injection of sterile saline 200 *μ*L 1 h before operation. The CLP-PD group was injected i.p. with the same volume of PD (15, 45, and 100 mg/kg, resp.) 1 h before CLP. The CLP-PD-ZnPP IX group was received an i.p. injection of the same volume of the mixed drugs of PD (100 mg/kg) and ZnPP IX (40 mg/kg) 1 h before CLP. ZnPP IX was dissolved in 0.2 M sodium hydroxide and added HCl to adjust pH to 7.4. The survivors were sacrificed at 24 h and blood and lung specimen were obtained for following analysis. The different doses of PD or ZnPP IX alone did not induce lung injury (data not shown).

### 2.4. Measurement of Serum Cytokines

The blood was enucleated at 24 h after CLP for serum TNF-*α* and IL-6 assay. The blood samples were centrifuged at 3000 rpm, 10 min. Then the serum was separated and transferred to other tubes and frozen at –20°C. Serum TNF-*α* and IL-6 were determined using commercially available ELISA kits according to manufacturer's recommendations.

### 2.5. Lung Wet-to-Dry Weight Ratio

The left lung was excised, washed in PBS, gently dried using blotting paper, and weighed. The tissue was then dried at 60°C for 72 h and reweighed. The change in the ratio of wet weight to dry weight was used as an indicator of lung edema formation.

### 2.6. Bronchoalveolar Lavage Fluid (BALF)

BALF was obtained by washing the airways three times with 1.5 mL of saline through a tracheal cannula. BALF was centrifuged at 4°C, 1500 g, for 10 min. The supernatant was harvested for total proteins analysis using the BCA protein assay kit and the pellet was smeared onto slides for cell classification and counting in BALF with a modified Giemsa stain.

### 2.7. Histopathology Analysis

The lung specimens were excised and fixed for one week in 10% formaldehyde at room temperature, and then embedded in paraffin. Serial paraffin sections (5 *μ*m) were stained with hematoxylin-eosin routinely for conventional morphological evaluation under a light microscope (Olympus, Tokyo, Japan). The severity of lung injury was judged by using a blind semiquantitative scoring system according to the following pathological features: (i) focal alveolar membrane thickening, (ii) capillary congestion, (iii) intra-alveolar hemorrhage, (iv) interstitial, and (v) intra-alveolar neutrophil infiltration. Each feature was scored from 0 to 3 based on its absence (0) or presence to a mild (1), moderate (2), or severe (3) degree, and a cumulative total histology score was determined [13].

### 2.8. Western Blotting Analysis

The protein of lung samples were prepared by the protein extract kit (20 mM Tris, 150 mM NaCl, 1 mM EDTA, 1 mM EGTA, 1% TritonX-100, 2.5 mM sodium pyrophosphate, 1 mM Na_3_VO_4_, 1 mM *β*-Glycerolphosphate, 1 *μ*g/mL leupeptin, and aprotinin). Protein concentrations were determined by BCA protein assay kit. 40 *μ*g protein extracts were fractionated on 12% polyacrylamide-sodium dodecyl sulfate (SDS) gel and then transferred to nitrocellulose membrane. The membrane was blocked with 5% (w/v) fat-free milk in Tris-buffered saline (TBS) containing 0.05% Tween 20, followed by incubation with a rabbit primary polyclonal antibody at 4°C overnight. Then the membrane was treated with horseradish peroxidase-conjugated goat anti-rabbit secondary antibody. Antibody binding was visualized with a chemiluminescence system and short exposure of the membrane to X-ray films (Kodak, Japan).

### 2.9. Assay of PGE_2_ and NO Production

PGE_2_ and NO production in lung tissues were measured by commercial kits according to the manufacturer's instructions. Briefly, lung tissues were thawed and homogenized, the tissue supernatants were collected. The amount of total protein in lung tissues was quantified by the BCA Protein assay kit. PGE_2_ was measured by an enzyme-linked immunosorbent assay (ELISA) using an antibody raised against mouse PGE_2_, absorbance was read at 450 nm. NO was analyzed with a nitrate/nitrite colorimetric assay. Firstly, nitrate was converted to nitrite by nitrate reductase, and 100 *μ*L of Griess reagent was added to 100 *μ*L tissue supernatants, which convert nitrite to a deep-purple azo compound. The absorbance of the latter was measured at 550 nm using a plate reader. PGE_2_ and NO concentration were normalized to the amount of total protein.

### 2.10. HO-1 Activity Assay

HO enzymatic activity was measured by bilirubin generation as described previously [14]. Briefly, frozen lung samples were homogenized in lysis buffer (250 mM Tris.HCl, pH 7.4; 150 mM NaCl; 250 mM sucrose; 0.5 mM PMSF; 1 *μ*g/*μ*L leupeptin; 1 *μ*g/*μ*L aprotinin). Microsomal fraction was obtained by successive centrifugations and washed with 0.15 M KCl followed by centrifugation (105,000 ×g for 30 min). The pellet was solubilized in 0.1 M potassium phosphate by sonication and stored at −80°C. The reaction was carried out in the mixture containing 2-3 mg/mL protein microsomal fraction; 1 mM glucose-6-phosphate; 0.2 unit/mL glucose-6-phosphate dehydrogenase; 0.8 mM NADPH; 0.025 mg/mL hemin at 37°C for 45 min. After chloroform extraction, the extracted bilirubin was calculated by the difference in absorbance between 464 and 530 nm. 

### 2.11. NF-*κ*B p65 Transcription Factor Assay

Nuclear protein extracts were obtained from lung tissues using a Nuclear Extract Kit (Active Motif, USA) according to the manufacturer's instructions. The p65 DNA binding activity was assessed by the TransAM NF-*κ*B Kit (Active Motif, USA) according to the manufacturer's instructions.

### 2.12. Statistical Analysis

The study data were expressed as mean ± standard deviation (SD) and differences between group means were calculated by One-way analysis of variance (ANOVA) and Student's *t*-test. Kaplan-Meyer survival curves were analyzed with the log-rank test. *P* values less than 0.05 were considered to be statistically significant.

## 3. Results

### 3.1. Effects of PD on CLP-Induced Lethality of Mice and Pathological Changes of Lungs

No death of mice was observed in the control group. When CLP was performed on mice, lethality of mice was 50% at 24 h and reached to 90% at 120 h. Pretreatment with PD dose-dependently decreased the lethality induced by CLP. However, the CLP-PD-ZnPP IX group had a higher mortality than the CLP-PD (100 mg/kg) group, which indicated a significant difference (*P* < 0.05); (Figure 1(b)).

**Figure 2:**
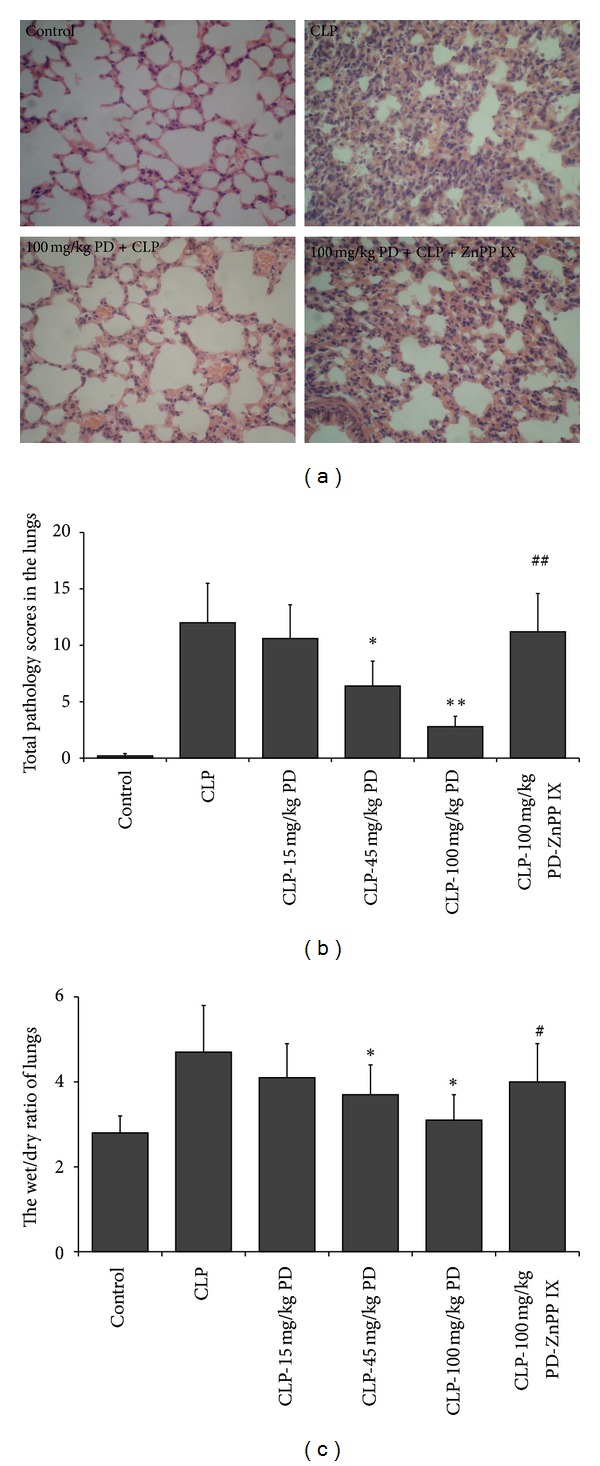
Histology changes and edema in the lungs from the treated-mice. Mice were pretreated i.p. with vehicle (PBS) or PD (15, 45, and 100 mg/kg, resp.) in the presence or absence of ZnPP IX (40 mg/kg) 1 h before CLP. The lung tissues were obtained from kill mice 24 h after CLP (a) Hematoxylin and eosin-staining (original magnification, 400x) and (b) total histology scores were determined. (c) The wet/dry ratio of lung tissue was assayed.  *P < 0.05,  **P < 0.01 versus the CLP group.  ^##^ *P* < 0.01 versus the CLP-PD group.

Because the lethality of septic mice is associated with acute lung injury, we examine the pathology of lung tissues by HE staining. In contrast to the structure of pulmonary alveoli in the control group, the alveoli in the CLP group were destructed, showing severe pathological abnormality including pulmonary interstitial hyperemia and edema, alveolar fluid exudation, and massive infiltration of inflammatory cells. These pathological changes were significantly alleviated by 100 mg/kg PD. However, the pathological injury of lung tissues in the CLP-PD-ZnPP IX group were exacerbated when compared with the CLP-PD (100 mg/kg) group, indicating HO-1 inhibitor ZnPP IX might abrogate the pulmonary protective effect of PD in septic mice (Figures 2(a) and 2(b)). In agreement with this pathological analysis, the wet/dry ratio of lung tissue showed a similar result (Figure 2(c)).

### 3.2. Effects of PD on Leukocytes Number and Total Protein in the BALFs

**Figure 3:**
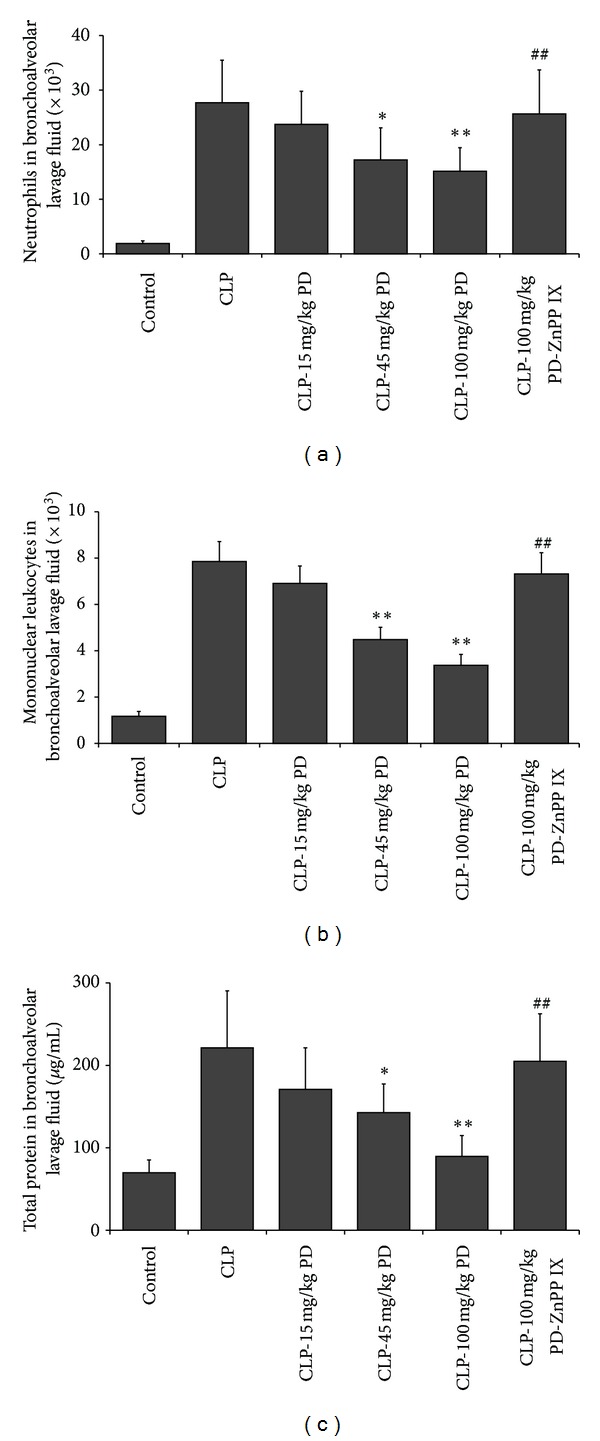
Effects of PD on leukocytes number and total protein in BALFs. Mice were pretreated i.p. with vehicle (PBS) or PD (15, 45, and 100 mg/kg, resp.) in the presence or absence of ZnPP IX (40 mg/kg) 1 h before CLP. At 24 h after CLP, BALF were collected for measurement of leukocytes ((a) and (b)) and total protein (c). Results are expressed as mean ± SD (*n* = 6).  *P < 0.05,  **P < 0.01 versus the CLP group.  ^##^ *P* < 0.01 versus the CLP-PD group.

To further access the extent of the airway and lung injury, we examined the amount of leukocytes and total proteins in the BALFs 24 h after CLP. The values were shown in Figures 3(a)–3(c). As expected, the CLP group had significantly higher levels of leukocytes and total protein in the BALF than the control group. Pretreatment mice with PD lowered infiltrating leukocytes and total proteins in the BALF especially at 100 mg/kg. However, ZnPP IX administration eliminated the inhibitory effects of PD on infiltration of leukocytes and total proteins in the BALFs of septic mice.

### 3.3. Effects of PD on Serum TNF-*α* and IL-6 Productions

**Figure 4:**
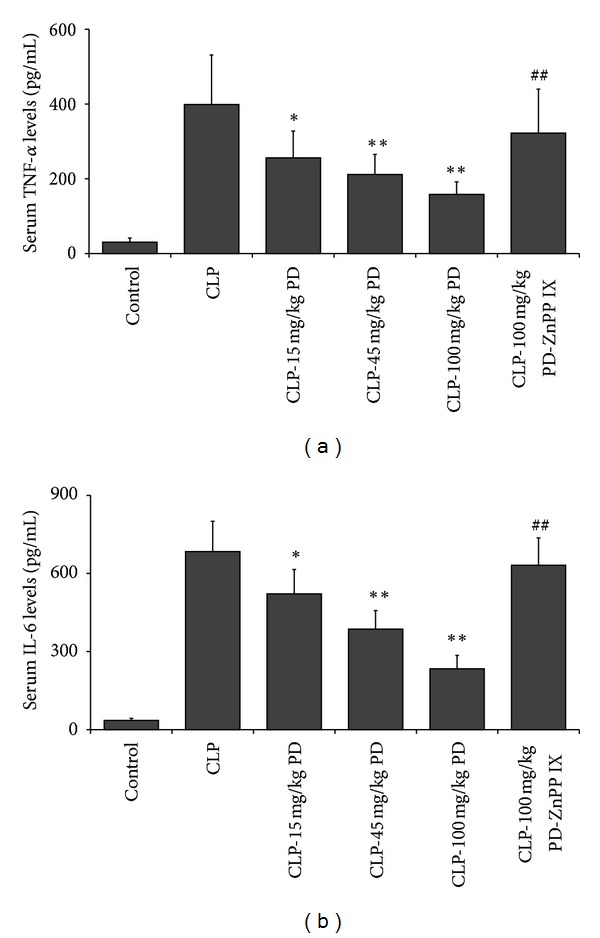
Effects of PD on the serum levels of TNF-*α* (a) and IL-6 (b) in mice. Mice were pretreated i.p. with vehicle (PBS) or PD (15, 45, and 100 mg/kg, resp.) in the presence or absence of ZnPP IX (40 mg/kg) 1 h before CLP. Serum were collected 24 h after CLP operation, the cytokines were assayed by ELISA. Results are expressed as mean ± S.D (*n* = 6).  *P < 0.05,  **P < 0.01 versus the CLP group. ^##^ *P* < 0.01 versus the CLP-PD group.

It has been shown that systemic proinflammatory cytokines have a central role in sepsis [15, 16]; we investigated next the effects of PD on CLP-induced systemic TNF-*α* and IL-6 production. As shown in Figure 4, the serum levels of TNF-*α* and IL-6 in the CLP group were remarkably increased as compared with these in the control group. However, the elevations of these proinflammatory cytokines were inhibited by pretreatment with PD in a dose-dependent manner. On the contrary, the inhibitory effects of PD on the CLP-induced TNF-*α* and IL-6 productions were blocked by ZnPP IX administration.

### 3.4. Effects of PD on COX-2 and iNOS Protein Expression, and PGE_2_ and NO Production in the Lungs

**Figure 5:**
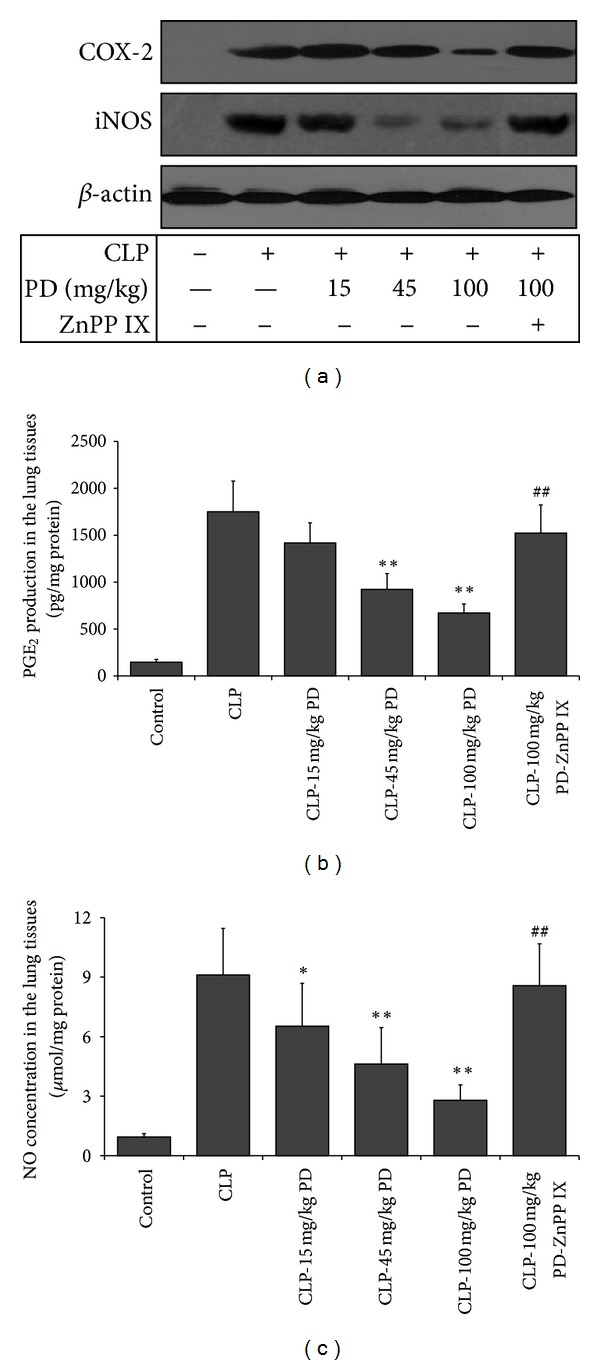
Effects of PD on the protein expression of COX-2 and iNOS (a), and PGE_2_ (b), and NO (c) production in the lungs of mice. Mice were pretreated i.p. with vehicle (PBS) or PD (15, 45, and 100 mg/kg, resp.) in the presence or absence of ZnPP IX (40 mg/kg) 1 h before CLP. The lung tissues were obtained 24 h after CLP for evaluation of COX-2, iNOS, PGE_2_, and NO. Results are expressed as mean ± SD (*n* = 6).  *P < 0.05,  **P < 0.01 versus the CLP group.  ^##^ *P* < 0.01 versus the CLP-PD group.

In comparison to the control group, in the CLP group, the lung COX-2 and iNOS protein expression were significantly elevated, pretreatment with PD dose-dependently suppressed these upregulated proteins. However, the CLP mice were pretreated with PD in combination of ZnPP IX, COX-2 and iNOS expressions in the lung tissues was blunted (Figure 5(a)). Similar results were observed in PGE_2_ and NO productions (Figures 5(b) and 5(c)).

### 3.5. Effects of PD on the Expression and Activity of HO-1 in the Lungs

**Figure 6:**
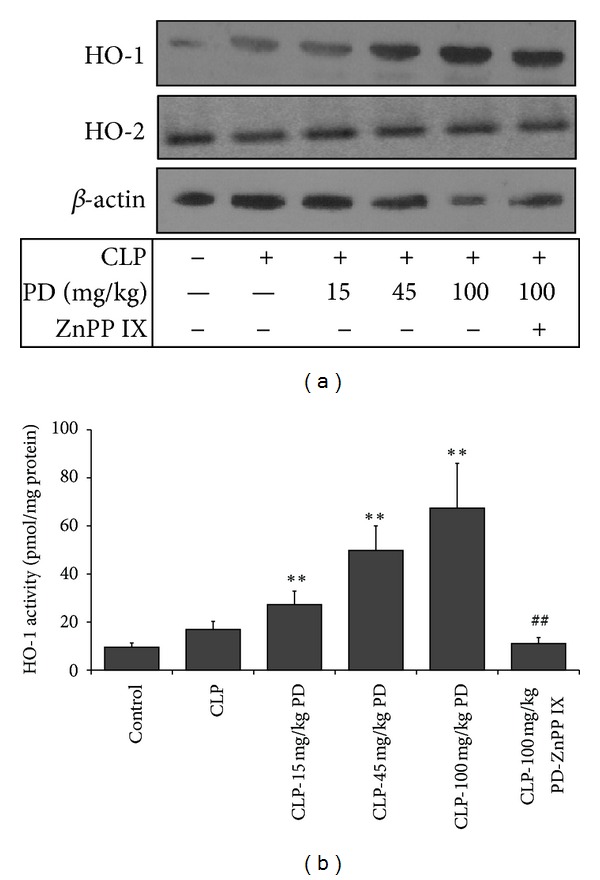
Effects of PD on the protein expression (a) and activity (b) of HO in the lungs of mice. Mice were pretreated i.p. with vehicle (PBS) or PD (15, 45, and 100 mg/kg, resp.) in the presence or absence of ZnPP IX (40 mg/kg) 1 h before CLP. The lung tissues were obtained 24 h after CLP for measurement of HO protein expression and activity. Results are expressed as mean ± S.D (*n* = 6).  *P < 0.05,  **P < 0.01 versus the CLP group.  ^##^ *P* < 0.01 versus the CLP-PD group.

 We also observed the change of HO-1, which has been regarded as an anti-inflammatory molecule [17]. Western blotting and activity analysis showed that the protein expression and activity of HO-1 in the lungs were not detected in the control group, but in the CLP-operated mice, HO-1 expression and activity had a slight increase. Pretreated with PD enhanced the expression and activity of HO-1 in lungs of septic mice in a dose-dependent manner. Further, the increase of HO-1 activity by PD was almost completely abolished by ZnPP IX administration, but HO-1 protein expression was not affected by ZnPP IX. However, no changes in HO-2 protein expression were observed in the lungs of all these groups (Figure 6).

### 3.6. Effects of PD on NF-*κ*B Signaling Pathway in the Lungs

**Figure 7:**
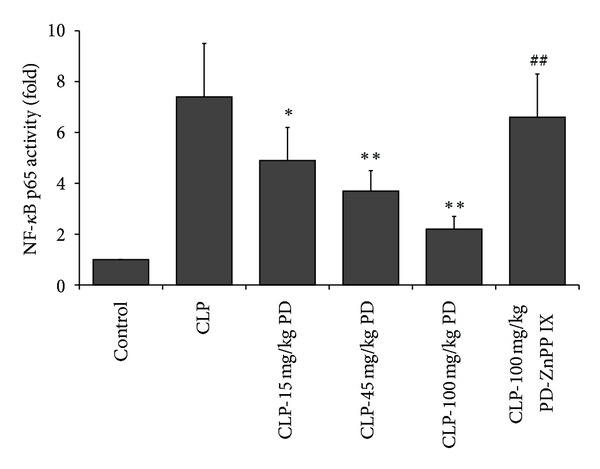
Effects of PD on the NF-*κ*B activation in mice. Mice were pretreated i.p. with vehicle (PBS) or PD (15, 45, and 100 mg/kg, resp.) in the presence or absence of ZnPP IX (40 mg/kg) 1 h before CLP, and after 24 h, nuclear protein extracts were obtained from lung tissues for analysis of NF-*κ*B activation. Results are expressed as mean ± S.D (*n* = 6).  *P < 0.05,  **P < 0.01 versus the CLP group.  ^##^ *P* < 0.01 versus the CLP-PD group.

The p65 is a main subunit of NF-*κ*B, which is activated to translocate to the nucleus. We used NF-*κ*B p65 transcription factor assay method to assay nuclear NF-*κ*B activity. As shown in Figure 7, in the CLP group, NF-*κ*B activity were markedly increased compared with the control group. However, PD pretreatment dose-dependently inhibited CLP-induced NF-*κ*B activity in the lung. Furthermore, the beneficial effect of PD in the NF-*κ*B activity of septic mice was abrogated by ZnPP IX.

## 4. Discussion 

In the present study, mice in the CLP model group have a high lethality and severe pathological lung injury including massive infiltration of inflammatory cells, hyperemia and edema of pulmonary interstitial. Pretreated mice with PD dose-dependently decreased CLP-induced lethality, accompanied with alleviated severe lung pathological injury and leukocytes number and total proteins in BALFs. Collectively, these results indicated that PD alleviated CLP-induced septic lung injury, which is in accordance with a previous study that PD had prophylactic and therapeutic effects on acute lung injury in rats with endotoxic shock [7].

In fact, sepsis is the intense systemic inflammatory response syndrome, which is associated with the overproduction of proinflammatory cytokines. These cytokines lead to recruitment of leukocytes, tissue damage, and multiple organ failure [18, 19]. Previous studies have indicated that proinflammatory cytokines especially TNF-*α* and IL-6 play crucial roles in septic lung injury [8, 20]. TNF-*α* has been also regarded as one of the major mediators of systemic progression and tissue damage in many severe diseases [21, 22]. An earlier study had proven that injecting TNF-*α* into experimental animals caused a syndrome that is indistinguishable from septic shock [16]. Neutralization of TNF-*α* could alleviate the development of sepsis in animal model [20]. In addition, previous studies indicated that the plasma level of IL-6 correlated with mortality in septic patients, and plasma levels of IL-6 might be used as a diagnostic marker for the presence of bacteremia. Antibody to block IL-6 improved further survival of mice in a bacterium-derived sepsis model [15, 23]. To determine whether these cytokines are associated with the protective effect of PD, we examined serum TNF-*α* and IL-6 levels in mice. We observed that PD decreased notably sepsis-induced serum TNF-*α* and IL-6 levels. These data supported that the protective effects of PD on septic lung injury in mice might be achieved by inhibition of these proinflammatory cytokines.

Apart from proinflammatory cytokines, some enzymes such as COX-2 and iNOS were also involved in the pathogenesis of sepsis [24]. COX-2 is an inducible enzyme in the conversion of arachidonic acid to inflammatory prostaglandins, which is usually absent or minimally present at baseline in most normal tissues but is highly induced in response to inflammatory stimuli [25]. Although some studies showed that COX-2 had anti-inflammatory actions, accumulating data suggested that the COX-2/PGE_2_ plays a vital role in augmenting inflammatory response and respiratory damage in sepsis [26, 27]. The different role of COX-2 might be associated with different types of inflammatory cells and prostaglandins during inflammatory process. Administration of NS-398, a specific COX-2 inhibitor, inhibited endotoxin-induced PGE_2_ and improved survival and restored leukocyte counts in septic mice [25, 28, 29]. Nitric oxide (NO), generated by the inducible nitric oxide synthase (iNOS), plays an important role in vascular tone, leukocyte rolling, and microvascular leakage [30]. iNOS has been identified in many cell types such as epithelial cells or alveolar macrophages and is activated in septic mice. Knockout of iNOS expression in mice drastically alleviated pulmonary inflammation in response to intravenous exposure to LPS. Furthermore, inhibition of iNOS activation significantly decreased the mortality and lung injury in septic mice [31–33]. Based on these data above, we determined these proteins expression in the lung by immunoblotting. Our results showed that PD was able to robustly inhibit the expression of iNOS and COX-2 protein mediated by CLP in a dose-dependent manner, which was in line with its protection on septic lung injury, indicating that the protective effects of PD on sepsis-induced lung injury might be by inhibiting COX-2/PGE_2_ and iNOS/NO pathways. 

NF-*κ*B is considered to an important transcriptional factor involved in the production of proinflammatory cytokines [24, 34]. Normally, NF-*κ*B is sequestered in an inactive form in the cytoplasm, when it is activated by inflammatory stimuli; NF-*κ*B translocates to the nucleus and subsequently initiates the transcription of inflammatory genes. Activation of the NF-*κ*B pathway has been implicated in the expressions of proinflammatory mediators, such as TNF-*α*, IL-6, COX-2, and iNOS. Increased activation of NF-*κ*B is found in alveolar macrophages, peripheral blood mononuclear cells, and neutrophils from patients with sepsis [35, 36]. A greater or more persistent nuclear accumulation of NF-*κ*B is associated with higher mortality and more persistent organ dysfunction, including pulmonary injury [37]. Therefore, inhibiting NF-*κ*B activation, which mediates the expression of proinflammatory mediators, appears to be a logical therapeutic target for controlling hyperinflammatory response including ALI. Thus, we confirmed whether PD could modulate the activation of these upstream signal molecules. We found that in parallel with the above results of proinflammatory mediators, PD also inhibited remarkably CLP-induced NF-*κ*B activation. These data suggested that the protective effects of PD on septic lung injury might be linked to inhibition of the activation of NF-*κ*B signaling pathway, resulting in suppression of proinflammatory mediators.

Alternatively, sepsis-induced inflammation is tightly regulated by some anti-inflammatory molecules that modulate the intensity of inflammation and promote its eventual resolution and return it back to homeostasis. Previous studies have demonstrated that HO-1 is pivotal for downregulating the incremented inflammatory process and maintaining homeostasis for the correct function of vital organs [17, 36]. To clarify the role of HO-1 in the protective effects of PD on septic lung injury, we first examined HO-1 protein expression and activity. Pretreatment CLP mice with increasing concentration of PD upregulated HO-1 but not HO-2 expression in the lungs in a dose-dependent manner. Further, we administered mice with ZnPP IX, the specific inhibitor of HO-1, to block HO-1 activity. Results indicated that ZnPP IX administration abrogated these benefits of PD not only in the mortality but also in the acute lung injury and inflammatory response. All these data suggested that the protective mechanisms of PD against septic lung injury might, at least partly, be by upregulation of HO-1. Previous studies indicated some transcription factors, as nuclear factor E2-related factor-2 (Nrf2), activator protein-1 (AP-1), and NF*κ*-B, are involved in this induction of HO-1 [38–40]. In this study, we found that CLP induced a slight increase in HO-1 expression and activity, although this increase had not a statistical significance compared with the control group. We deduced that HO-1 upregulation in the CLP group might be by NF*κ*-B activation. However, it is impossible that upregulation of HO-1 by PD is mediated by NF*κ*-B pathway, since PD inhibited CLP-induced NF*κ*-B activation. Recent studies show that PD has obvious antioxidant property thought increasing the activities of superoxide dismutase (SOD) and catalase (CAT) [41, 42]. In fact, Nrf2 is an important regulator against oxidative stress, which increases the expression of HO-1, SOD, and CAT [43]. Thereby, we speculate that PD may be through the mechanism of Nrf2 activation to upregulation of HO-1. Certainly, to verify this hypothesis, further investigation needs to be done.

To conclude, we have demonstrated that PD could effectively exert protective roles against CLP-induced septic lung injury, which alleviated lung pathological injury, reduced proinflammatory mediators, and attenuated lung inflammatory responses. Such protective effects were probably carried out through suppression of NF-*κ*B signaling pathways. Further, these effects appear to be mediated through upregulating HO-1. Therefore, it is expected that PD might contribute to delaying ALI progression and prolonging life in patients with lung damage.
